# Propofol Infusion Syndrome: A Rare Complication From a Common Medication

**DOI:** 10.7759/cureus.31940

**Published:** 2022-11-27

**Authors:** Tutul Chowdhury, Ashish Thapa, Nevil Kadakia, Nabina Khadka, Nicole Gousy

**Affiliations:** 1 Internal Medicine, Interfaith Medical Center, Brooklyn, USA; 2 Medicine, American University of Antigua, New York City, USA

**Keywords:** rhabdomyolysis with acute renal failure, creatinine kinase, cellular and molecular biology, propofol infusion syndrome, propofol based sedation

## Abstract

Propofol infusion syndrome (PRIS) is a multifactorial condition that, upon propofol administration, can interrupt critical cellular processes. This can lead to cellular damage that translates as multi-organ system failure that has the potential to be life-threatening. Due to the rarity of this condition, we report a case of PRIS in a 46-year-old male to help bring awareness to this severe condition caused by a relatively common medication. This patient was brought in due to unresponsiveness secondary to multi-substance abuse and respiratory disease and initially had elevated creatinine kinase levels that eventually subsided with appropriate management. However, after prolonged infusion of propofol, his creatinine kinase levels began to drastically rise, alluding to the development of propofol infusion syndrome. Once the offending agent was discontinued, the patient's creatinine kinase levels once again began to normalize.

## Introduction

Propofol infusion syndrome is a rare condition characterized by the triad of lactic acidosis, rhabdomyolysis, and cardiovascular collapse following a high infusion of propofol over prolonged periods of time [1]. The pathophysiology of this condition includes mitochondrial impairment resulting in the disruption of fatty acid beta-oxidation, the electron transport chain, and dysfunction of beta-adrenoceptors and cardiac calcium channels [2]. Our patient is a 46-year-old male who was admitted to our hospital for acute respiratory failure, septic shock, and rhabdomyolysis. The patient was managed in the intensive care unit (ICU) with mechanical ventilation, standardized propofol and fentanyl for sedation and analgesia, respectively, broad-spectrum antibiotics and intravenous (IV) fluids, and vasopressors. This management resulted in a favorable improvement of his rhabdomyolysis as indicated by an initial decrease in creatinine kinase (CK) from 2931 to 891. On hospital day five, however, his CK worsened to 27,467, along with triglycerides (TGA) increasing from 70 to 211, with worsening liver enzymes without significant impact on renal function or acid-base disturbances.

## Case presentation

We present a 46-year-old male with a past medical history of asthma and substance drug abuse. He was brought in by emergency medical services (EMS) when his sister found him unresponsive on the floor, having difficulty breathing. Due to his lack of consciousness, a review of systems could not be obtained. His last known admission was three years ago due to an asthma exacerbation; since then, his asthma has been well-controlled with albuterol. He has never been intubated and has no known allergies. Family members report the patient frequently using illicit opioids for more than five years. Family history is significant for hypertension and asthma in the sister.

**Figure 1:**
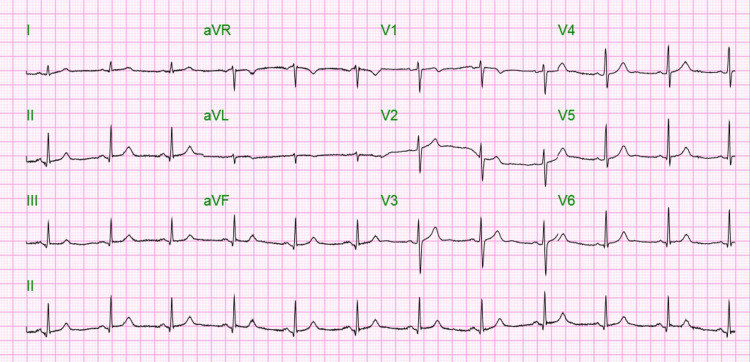
EKG showed heart rate of 69, QTc of 411, normal sinus rhythm

**Figure 2:**
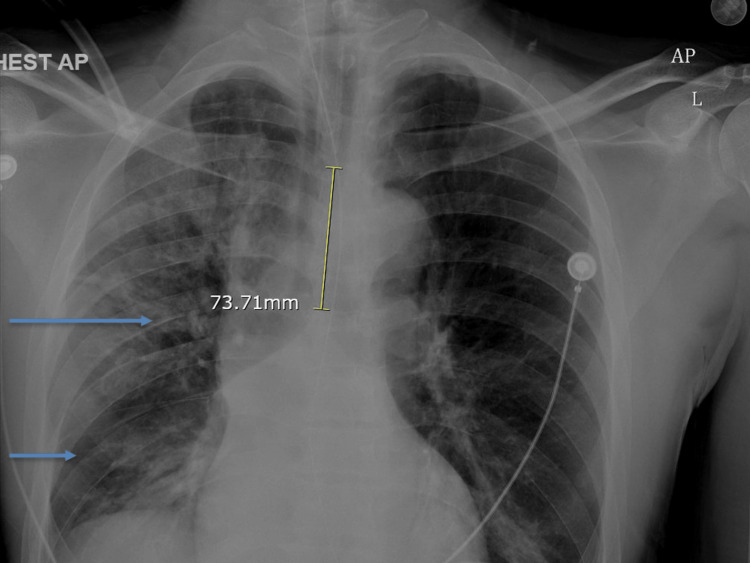
Chest X-ray showing airspace opacities in the right mid and lower lung (arrows)

**Figure 3:**
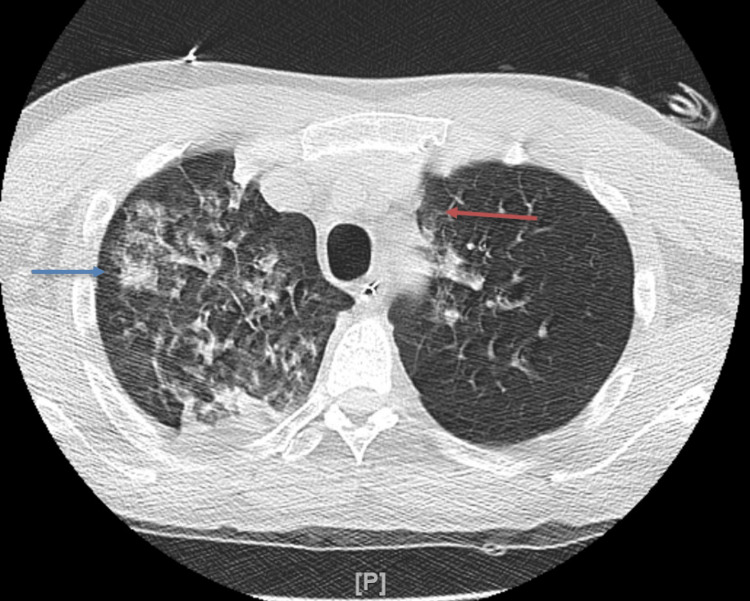
CT of the chest CT Chest revealed extensive pulmonary parenchymal findings detailed above including consolidation of the right lower lobe (blue arrow) which was considered a combination of both atelectasis and infection. Mediastinal lymphadenopathy (red arrow) was noted in addition to axillary lymphadenopathy

**Figure 4:**
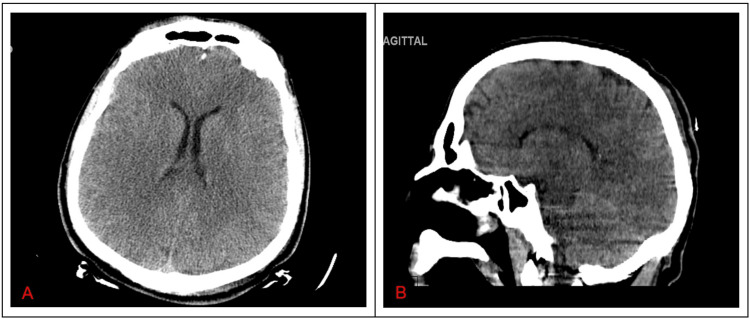
A CT of the head was negative for acute changes (A and B)

Upon arrival to the emergency department, the patient was in obvious respiratory distress. Triage vitals at that time showed a temperature of 101.4 F, pulse of 128 beats per minute, respiratory rate of 26 breaths/minute, blood pressure of 159/84 mmHg, and saturating at 62% in a non-rebreather mask. An eye examination illustrated pinpoint pupils that were nonreactive to light. A cardiopulmonary exam revealed bilateral wheezing in the lung bases with tachypnea and tachycardia with normal S1 and S2 sounds without murmurs. Further examination revealed a soft abdomen with active bowel sounds without distention appreciated. Additionally, multiple patches of hyperpigmentation were identified on both legs. An evaluation of the patient's central nervous system could not be done due to subsequent sedation with propofol and intubation with mechanical ventilation via pressure-regulated volume control (PRVC) mode (400mL tidal volume/50cmH20/7 positive end-expiratory pressure /100% FiO2) on arrival to the emergency department. Appropriate labs were completed upon arrival in addition to an electrocardiogram (ECG), a chest X-ray, and a computerized tomography (CT) scan (Tables 1-7; Figures 1-3). A head CT was also performed to rule out any possible head trauma in light of finding the patient with a loss of consciousness on the floor (Figure 4).

**Table 1:** Results of the patient's complete blood count WBC - white blood cells; RBC - red blood cells; MCV - mean corpuscular volume; MCH - mean corpuscular hemoglobin; MCHC - mean corpuscular hemoglobin concentration; RDW - red blood cell distribution width; MPV - mean platelet volume

Component	Reference range and units	Result
WBC	4.5 - 11.0 10x3/uL	14.6 High
RBC	4 - 5.7 10x6/uL	4.40
Hemoglobin	13.0 - 17.0 g/dL	12.2 Low
Hematocrit	39 - 53 %	37.2 Low
MCV	80 - 100 fL	84.4
MCH	26.0 - 33.0 pg	27.7
MCHC	30.5 - 36.0 g/dL	32.8
RDW	11.5 - 15.1 %	14.3
MPV	7.0 - 11.5 fL	9.7
Neutrophils %	40.0 - 70.0 %	83.2 High
Lymphocytes %	22.0 - 48.0 %	4.9 Low
Monocytes %	2.0 - 14.0 %	11.7
Eosinophils %	0.5 - 5.0 %	0.0 Low
Basophils %	0.0 - 2.0 %	0.2
Neutrophils absolute	2.00 - 7.90 10x3/uL	12.20 High
Lymphocytes absolute	1.00 - 4.80 10x3/uL	0.70 Low
Monocytes absolute	0.30 - 1.00 10x3/uL	1.70 High
Eosinophils absolute	0.00 - 0.40 10x3/uL	0.00
Basophils absolute	0.00 - 0.10 10x3/uL	0.00
Platelets	130 - 400 10x3/uL	26

**Table 2:** Results of the patient's complete metabolic panel ALT - alanine aminotransferase; AST - aspartate aminotransferase; eGFR - estimated glomerular filtration rate

Component	Reference range and units	Result
Blood urea nitrogen	8.9 - 20.6 mg/dL	29.8 High
Creatinine	0.72 - 1.25 mg/dL	1.45 High
Sodium	136 - 145 mmol/L	150 High
Potassium	3.5 - 5.1 mmol/L	4.2
Chloride	98 - 107 mmol/L	116 High
CO2	22 - 29 mmol/L	21 Low
Calcium	8.4 - 10.2 mg/dL	9.4
Anion gap	mmol/L	13
Protein, total	6.0 - 8.3 g/dL	9.0 High
Albumin	3.5 - 5.2 g/dL	4.2
Bilirubin, total	0.2 - 1.2 mg/dL	0.8
ALT	10 - 55 U/L	11
AST	5 - 34 U/L	50 High
Alkaline phosphatase	40.0 - 150.0 U/L	50.2
Albumin/globulin ratio		0.9
eGFR	>=90.0 mL/min/1.73m2	60.2 Low

**Table 3:** Patient's coagulation panel with inflammation markers BNP - brain natriuretic peptide; CRP - C-reactive protein; PT - prothrombin time; INR - internationalized normalized ratio; PTT - partial thromboplastin time

Component	Reference range and units	Result
BNP	10.0 - 100.0 pg/mL	89.9
Lactate	0.50 - 1.90 mmol/L	2.85
Creatine kinase	30.0 - 200.0 U/L	2931.7
CRP	0.50 - 1.00 mg/dL	5.80
Troponin	0.0 - 35.0 ng/L	22.4
PT	9.8 - 13.4 sec	14.9
INR	0.85 - 1.15	1.31
PTT	24.9 - 35.9 sec	37.9
Vitamin D	30.0 - 100.0 ng/mL	45.8

**Table 4:** Patient's urinalysis UA - urinalysis

Component	Reference range and units	Result
Color, UA	Light yellow, yellow	Yellow
Clarity, UA		Clear
Specific gravity, UA	1.005 - 1.030	1.015
pH, UA	5.0 - 8.0	6.0
Protein, UA	Negative mg/dL	100 Abnormal
Glucose, UA	Negative mg/dL	Negative
Ketones, UA	Negative mg/dL	15 Abnormal
Bilirubin, UA	Negative	Negative
Blood, UA	Negative	Large Abnormal
Nitrite, UA	Negative	Negative
Urobilinogen, UA	0.2 - 1.0 EU/dl	0.2
Leukocytes, UA	Negative	Negative

**Table 5:** Patient's microscopic urinalysis WBC - white blood cells; RBC - red blood cell; US - urinalysis; HPF - high power field

Component	Reference range and units	Result
WBC, UA	0 - 5 /HPF	0-5
RBC, UA	0 - 4 /HPF	0-4
Bacteria, UA	None /HPF	Moderate Abnormal
Squamous Epithelial Cells, UA	0 - 4.0 /HPF	0-4.0
Renal Epithelial Cells, UA	0 - 4 /HPF	4-10 Abnormal
Hyaline Casts, UA	0 - 2 /LPF	2-5 Abnormal

**Table 6:** Patient's viral panel results PCR - polymerase chain reaction; NAA - nucleic acid amplification

Component	Reference range and units	Result
SARS-COV-2 PCR by NAA	Not detected	Not detected
Influenza A, NAA	Not detected	Not detected
Influenza B, NAA	Not detected	Not detected

**Table 7:** Patient's ABG results showing hypoxia ABG - arterial blood gases

Component	Reference range and units	Results
pH, arterial	7.35-7.45	7.33
pCO2, arterial	35-45 mm hg	47.4
pO2, arterial	80-100 mm hg	197
HCO3, arterial	22-28 mmol/L	24.3
Base excess, arterial	-3.2-1.8 mmol/L	-0.9
O2 saturation, arterial	92-98.5%	98.8
Carboxyhemoglobin	0.5-1.5%	0.7
Total CO2, arterial	19=25.0 mmol/L	25.7
Normal Aa gradient	Mm Hg	14

He was started on broad-spectrum antibiotics, including vancomycin, meropenem, and doxycycline. He received an initial 2-liter bolus of normal saline, which resulted in the normalization of his lactic acid. Intravenous fluid with 0.9% normal saline was continued for hydration of the kidney, and a Foley catheter was inserted for close monitoring of input and output charting.

**Figure 5:**
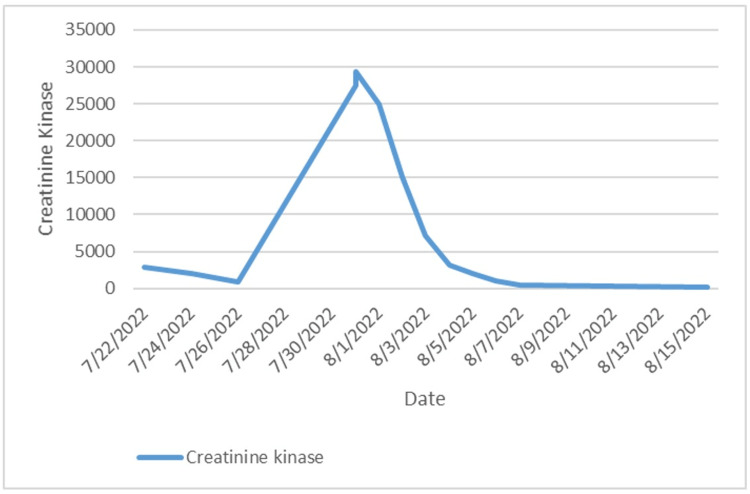
A graph showing the trending creatinine kinase levels of the patient during the course of their admission. On 08/01/2022, propofol administration was discontinued

On the day of admission, the patient's creatine kinase (CK) was 2931.7, with urinalysis showing blood in large amounts. Sepsis secondary to aspiration pneumonia from drug intoxication was suspected based on positive levels of cocaine, opiates, and benzodiazepines on urinalysis on admission. Acute respiratory failure was also considered at this time. After intubation, a propofol infusion was commenced for seven days. During routine lab testing, a sustained trend in increasing CK levels was observed, with a maximum value of >29,000. Since elevated CK levels were observed after the initiation of propofol infusion, rhabdomyolysis secondary to prolonged propofol infusion was considered; therefore, propofol was discontinued. This resulted in a sustained down trending of CK values (Figure 5).

## Discussion

Propofol infusion syndrome (PRIS) is a rare but potentially fatal condition due to propofol infusion. The term 'PRIS' was first described by Bray in the pediatric population in the 1990s and later reported in adults by Marinella [1,2]. PRIS is multifactorial, with major contributing risk factors including prolonged use (usually > 48 hours) and high-dose infusions (4 mg/kg/hr). Critical illness, carbohydrate depletion in cases like starvation, liver diseases and malnutrition, advanced stress level, subclinical mitochondrial disease, and use of corticosteroids and vasopressors can act as triggering factors [3,4]. The true incidence of PRIS is still not known. However, a study by Hwang et al. showed an incidence of PRIS around 17% in propofol doses of at least 5 mg/kg/hour and around 31% in doses > 6 mg/kg/hour [5]. In 2009, another study done by Roberts et al. showed that 1,017 critically ill patients on propofol for longer than 24 hours reported an incidence of PRIS to be approximately 1.1%, while an analysis by Fong et al. estimated an incidence of approximately 30% [2,6,7].

Propofol affects oxidative phosphorylation in the mitochondrial electron transport chain. It also inhibits carnitine palmitoyl transferase I causing defects in fatty acid metabolism. This causes discrepancies in energy demand and utilization, leading to disruptions in normal cellular function [8,9]. Propofol also inhibits β-adrenergic receptor and calcium channel binding, leading to cardiac dysfunction [10]. Defects in normal aerobic respiration lead to lactic acidosis, which can further lead to rhabdomyolysis with subsequent kidney injury. The major clinical presentations of PRIS include high anion gap metabolic acidosis, rhabdomyolysis, acute refractory bradycardia, and a variety of arrhythmias, including right bundle branch block, atrial fibrillation, supraventricular and ventricular tachycardia eventually causing asystole. Additionally, lipemia, hepatomegaly, hyperkalemia, and renal failure can also be appreciated in PRIS [2,3].

In our case, the patient displayed many of the diagnostic criteria for PRIS, including rhabdomyolysis resulting in acute kidney injury, elevated lactate levels, and a high anion gap metabolic acidosis. The most important preexisting risk factor was sedation with propofol for more than 48 hours, which resulted in significant elevations in CK, an indicator of muscle injury. Since the CK levels initially decreased from admission levels upon administration of adequate IV hydration, we can interpret the sudden increasing levels of CK as a result of prolonged propofol. The decrease in CK levels when propofol was discontinued also alludes to PRIS being the cause of this episode. 

While discussing and managing possible PRIS, we need to keep broad differentials. Hypoperfusion leading to shock mechanism, medications (including beta-agonist, vasoactive drugs, and antiviral drugs), and tissue ischemia should also be accounted for the elevation of lactic acid. Congenital conditions like Brugada syndrome should be evaluated. Prolonged immobilization, recent traumatic events, seizure disorder, and recent contrast exposure may lead to rhabdomyolysis and, ultimately, renal complications. Lactic acid elevation will cause high anion gap metabolic acidosis (HAGMA) and may further worsen hyperkalemia. First-line management is to discontinue propofol and use other sedatives if needed. Regular monitoring of lab reports, including CK and TGA levels, avoidance of prolonged propofol therapy, and higher dose infusion is the best for ideal management. Hyperkalemia, acute renal failure, and cardiovascular complications should be managed accordingly.

## Conclusions

PRIS is a fatal condition caused by the cellular interruption of aerobic respiration upon propofol administration. The incidence of this condition is still unknown; however, it is thought to be induced by increased doses or prolonged administration of this medication. This can lead to severe consequences that affect every bodily system and can be fatal. In this case presentation, we report a male with severe kidney failure that resolved when propofol was discontinued. We report this case to bring to light the relatively rare complication of propofol and to increase awareness and clinical suspicion in a deteriorating patient on propofol.
